# Uncovering the Impact of Lymphadenectomy in Advanced Gastric Cancer: A Comprehensive Review

**DOI:** 10.3390/life13081769

**Published:** 2023-08-18

**Authors:** Venera-Cristina Dinescu, Veronica Gheorman, Eugen Florin Georgescu, Ștefan Paitici, Marius Bică, Ștefan Pătrașcu, Marius Gabriel Bunescu, Romeo Popa, Mihaela Corina Berceanu, Ana Maria Pătrașcu, Lavinia Maria Gheorman, Sorin Nicolae Dinescu, Ion Udriștoiu, Victor Gheorman, Mircea Cătălin Forțofoiu, Tiberiu-Ștefăniță Țenea Cojan

**Affiliations:** 1 Department of Health Promotion and Occupational Medicine, University of Medicine and Pharmacy of Craiova, 200349 Craiova, Romania; venera.dinescu@umfcv.ro; 2 Department of Cardiology, University of Medicine and Pharmacy of Craiova, 200349 Craiova, Romania; mihaela.berceanu@umfcv.ro; 3 Department of Surgery, University of Medicine and Pharmacy of Craiova, 200642 Craiova, Romania; eugenyok@yahoo.com (E.F.G.); marius.bică@umfcv.ro (M.B.); stef.patrascu@gmail.com (Ș.P.);; 4 Occupational Medicine Department, University of Medicine and Pharmacy of Craiova, 200349 Craiova, Romania; marius.bunescu@umfcv.ro; 5 Department of Pharmacology, University of Medicine and Pharmacy of Craiova, 200349 Craiova, Romania; romeo_rop@yahoo.com; 6 Hematology Department, University of Medicine and Pharmacy of Craiova, 200349 Craiova, Romania; 7 Department of Diabetology, University of Medicine and Pharmacy of Craiova, 200349 Craiova, Romania; lmgheorman@yahoo.com; 8 Department of Epidemiology, University of Medicine and Pharmacy of Craiova, 200349 Craiova, Romania; sorin.dinescu@umfcv.ro; 9 Department of Psychiatry, University of Medicine and Pharmacy of Craiova, 200349 Craiova, Romania; ion.udristoiu@umfcv.ro (I.U.); victor.gheorman@umfcv.ro (V.G.); 10 Internal Medicine Department, University of Medicine and Pharmacy of Craiova, Filantropia Hospital of Craiova, 200143 Craiova, Romania; catalin.fortofoiu@umfcv.ro

**Keywords:** gastric cancer, lymphadenectomy, surgical treatment, survival, complications

## Abstract

Gastric cancer is a significant health concern worldwide, and lymphadenectomy plays a crucial role in its treatment. However, there is ongoing debate regarding the optimal approach—D1 or D2 lymphadenectomy. This paper aims to synthesize the available evidence by conducting a comprehensive literature review and comparing the advantages and disadvantages of both techniques. The analysis includes studies, clinical trials, and systematic reviews that assess survival outcomes, morbidity, and quality of life. The selected studies revealed different outcomes associated with D1 and D2 lymphadenectomy, including lymph node harvest, disease control, recurrence rates, and overall survival. Postoperative complications also varied between the two techniques. These findings highlight the complex considerations involved in selecting the most suitable lymphadenectomy approach for individual patients. Therefore, the decision requires an individualized assessment that considers the potential benefits and risks of D1 and D2 techniques. A collaborative approach involving interdisciplinary teams is crucial for developing personalized treatment plans that optimize both oncological outcomes and postoperative quality of life.

## 1. Introduction

Cancer is a significant public health issue worldwide and is the second leading cause of death globally, with approximately 10 million annual deaths attributed to this disease [1,2,3,4,5]. In 2020, there were 10.3 million newly diagnosed cases of cancer and 9.8 million cancer-related deaths reported worldwide [2,3]. Of these cases, gastric cancer was the fourth most common neoplasm in both genders, with 1.0 million newly diagnosed occurrences and 0.7 million cancer-related deaths reported in 2020 [2,4].

The prevalence of advanced gastric cancer varies by geographic location and population [5]. In Europe and North America, the incidence and mortality rates have decreased over the past few decades, but they remain high in Asia and parts of South America [6].

According to GLOBOCAN 2020 statistics, the estimated incidence of stomach cancer in Romania is 3026 new cases per year [7]. Stomach cancer is the fifth most common cancer in Romania, accounting for 5.1% of all cancer cases. It is also the fourth most common cause of cancer-related death, with an estimated 2379 deaths annually [8].

Gastric carcinoma primarily affects the elderly population, often being diagnosed in individuals aged 65 years and older. However, recent studies have reported a concerning rise in the incidence of gastric cancer among younger age groups, indicating a shifting trend in the demographics of this disease.

Advanced gastric cancer is a complex and multifactorial disease characterized by the proliferation and metastasis of abnormal cells originating in the stomach [7]. There are various risk factors associated with the development of advanced gastric cancer [8,9,10,11,12,13]. The most common risk factors include Helicobacter pylori infection, age, family history of gastric cancer, smoking, diet, obesity and previous stomach surgery. Chronic inflammation caused by H. pylori infection can lead to the development of cancer, which is more common in older adults and those with a family history of gastric cancer. Additional significant risk factors for gastric cancer include tobacco use; diets rich in smoked, pickled, salted, or preserved foods and low in fruits and vegetables; as well as obesity. Additionally, people who have had surgery to remove part of their stomach are at increased risk of developing gastric cancer. It is essential to identify and manage these risk factors to reduce the incidence of advanced gastric cancer. 

These risk factors contribute to the development of gastric cancer by causing chronic inflammation in the stomach lining, which can lead to damage to the DNA in cells. Over time, this damage can accumulate and lead to the development of cancer. Moreover, some risk factors, such as smoking and obesity, can promote the growth of cancer cells by increasing inflammation and altering hormone levels in the body.

Some of the common symptoms of advanced gastric cancer include persistent abdominal pain, nausea, vomiting, fatigue, and significant weight loss [14]. However, these symptoms may be vague and sometimes go unnoticed until the cancer has advanced to a later stage. Unfortunately, most cases of advanced gastric cancer are diagnosed in their later stages when treatment options become limited and the prognosis is poor [15,16,17,18,19]. 

This article aims to delve into the use of lymphadenectomy in advanced gastric cancer, specifically exploring the efficacy of D1 versus D2 lymphadenectomy in patients. Through an in-depth analysis of this topic, the article seeks to shed light on the benefits and drawbacks of both methods, providing insights into the most effective approach to treating advanced gastric cancer. The article aims to provide a comprehensive overview that informs medical professionals and researchers in their treatment and research efforts.

## 2. Materials and Methods

To achieve this, we aimed to identify various types of studies, including retrospective studies, meta-analyses, and randomized controlled trials, to gather a comprehensive understanding of the topic (the flow diagram can be find in Supplementary File). The search strategy employed comprehensive databases, including PubMed, Embase, and Cochrane Library, using specific keywords and criteria to identify eligible studies. Inclusion and exclusion criteria were applied to screen the identified studies, and data extraction was performed using a standardized approach. Subgroup analyses were performed to examine potential heterogeneity and evaluate the robustness of the findings. By following this comprehensive approach, the review uncovers essential evidence and provides valuable insights that can assist clinicians and researchers in making informed decisions regarding the optimal lymphadenectomy approach for patients with advanced gastric cancer.

Our search strategy aimed to identify relevant studies on the efficacy of D1 versus D2 lymphadenectomy in advanced gastric cancer. We utilized the following keywords: ‘lymphadenectomy’, ‘advanced gastric cancer’, ‘D1’, ‘D2’, ‘surgical treatment’, ‘survival’, ‘complications’, ‘meta-analysis’, and ‘randomized controlled trials’. We used a combination of these keywords in our search queries, employing Boolean operators, such as ‘AND’ and ‘OR’, to identify studies that met our inclusion criteria. Additionally, we reviewed the reference lists of relevant articles to identify any additional studies that might have been missed. A risk of bias assessment was conducted as part of our comprehensive review process. We followed the guidelines and recommendations provided by the Cochrane Collaboration’s tool for assessing risk of bias. This assessment involved examining potential biases in key domains, including selection bias, performance bias, detection bias, attrition bias, reporting bias, and other sources of bias.

Upon completion, each included study was evaluated, and the risk of bias for each domain was categorized as “low risk”, “high risk”, or “unclear risk”. This comprehensive assessment allowed us to thoroughly investigate the quality and internal validity of the included studies and provided valuable insights for the interpretation of our findings. This comprehensive search strategy allowed us to identify a range of studies that provided evidence on the topic of interest. 

The inclusion criteria applied in the screening process for selecting studies were as follows:

- Studies conducted on patients diagnosed with advanced gastric cancer (stage II or higher);
- Comparison of D1 and D2 lymphadenectomy techniques;
- Availability of data on patient outcomes, such as overall survival, disease-free survival, and postoperative complications;
- Published in the English language;
- Full-text articles or abstracts available.

On the other hand, the exclusion criteria aimed to eliminate studies that did not meet specific requirements:

- Case reports, letters, editorials, or conference abstracts;
- In vitro or animal studies;
- Studies with insufficient data or lack of direct comparison between D1 and D2 lymphadenectomy;
- Studies with incomplete or unclear methodology.

To ensure the inclusion of recent and up-to-date research, we limited our search to studies published within the past 10 years, i.e., from 2012 to 2022. These inclusion and exclusion criteria were applied during both the initial screening of study titles and abstracts, and the subsequent full-text assessment. The goal was to ensure that only relevant and high-quality studies were included in the review, thereby enhancing the validity and reliability of the findings.

The PICOS statement for this study was as follows: 

Population: This study focused on patients diagnosed with advanced gastric cancer, specifically those in stage II or higher. 

Intervention: The primary intervention being investigated was the comparison of the D1 and D2 lymphadenectomy techniques. 

Comparison: The study aimed to compare the outcomes and efficacy of the D1 and D2 lymphadenectomy techniques in patients with advanced gastric cancer. 

Outcome: The study examined various patient outcomes, including overall survival, disease-free survival, and postoperative complications, to evaluate the effectiveness of the different lymphadenectomy techniques. 

Study design: The study design encompassed a comprehensive literature review with a specific focus on retrospective studies, meta-analyses, and randomized controlled trials. These sources were selected to provide a robust evidence base for assessing the comparative effectiveness of the D1 and D2 lymphadenectomy techniques in advanced gastric cancer treatment.

## 3. Discussion and Results 

### 3.1. Lymphadenectomy for Gastric Cancer

Lymphadenectomy is a surgical procedure used for removing lymph nodes near a cancerous tumor that is commonly performed for gastric cancer treatment [20,21]. Its purpose is to eliminate any potential cancer cells that might have spread to the lymph nodes [22]. This is crucial because cancer cells can easily migrate through the lymphatic system and create new tumors in other body parts. By removing the lymph nodes, the chances of cancer recurrence decrease.

Lymphadenectomy is a complex surgical procedure that demands careful planning and execution. The surgeon needs to identify the precise location of the lymph nodes that must be removed and ensure the procedure’s safety. It can be performed either through traditional open surgery or minimally invasive techniques like laparoscopy.

### 3.2. Lymphadenectomy Extent in Relation to Gastrectomy Type

Lymphadenectomy extent refers to the range of lymph nodes that are removed during a surgical procedure. In the context of gastrectomy, the extent of lymphadenectomy is determined based on the type of gastrectomy being performed. There are different classification systems used to define lymphadenectomy extent, with the most commonly used being the Japanese Gastric Cancer Association (JGCA) and the American Joint Committee on Cancer (AJCC) systems.

According to the JGCA classification, lymphadenectomy is categorized into several levels: D0, D1, D1+, D2, D2+, and D3. D0 refers to no lymphadenectomy, while D1 involves the removal of perigastric (stations 1, 3, 4, and 7) and proximal (station 2) lymph nodes. D1+ includes additional lymph nodes, such as the left gastric (station 7), right gastric (station 5), and splenic hilar (station 10) lymph nodes [22]. D2 involves the removal of extended lymph node stations, including those in the omental bursa (stations 8a and 9), along the common hepatic artery (stations 8a and 12a), and around the celiac trunk (stations 9 and 11), among others. D2+ includes additional nodes, such as the left gastric artery (station 7), splenic (stations 10 and 11p), and hepatic (stations 11p and 12a) lymph nodes. D3 is the most extensive level, encompassing the D2 stations as well as lymph nodes along the splenic artery (stations 11d and 10); these nodes are commonly referred to as para-aortic nodes.

The decision regarding the extent of lymphadenectomy is influenced by multiple factors, including tumor characteristics, patient factors, and surgeon expertise.

- Tumor Characteristics: The location, size, depth of invasion, histology, and stage of the gastric cancer play a crucial role in determining the appropriate extent of lymphadenectomy. Aggressive lymphadenectomy is often recommended for more advanced stages of gastric cancer, as it helps in achieving better disease control.
- Patient Factors: Patient-related factors, such as age, overall health status, comorbidities, and fitness for surgery, need to be taken into consideration. Lymphadenectomy has associated risks, including increased operative time and potential postoperative complications, which need to be weighed against the potential benefits for each individual patient.
- Surgeon Expertise: The experience and proficiency of the surgical team in performing lymphadenectomy at different levels also contribute to the decision-making process. D3 lymphadenectomy is technically demanding and requires expertise in handling complex anatomical structures.
- Shared Decision Making: Ultimately, the decision regarding the extent of lymphadenectomy should be made through shared decision making between the patient, surgeon, and multidisciplinary team. The risks and benefits of each level of lymphadenectomy must be discussed with the patient, considering individual tumor characteristics and patient factors, to determine the most appropriate approach for achieving optimal oncologic outcomes while minimizing complications. Surgeons with expertise in advanced gastric cancer surgery and lymphadenectomy play a critical role in guiding patients through this decision-making process.

### 3.3. Implications of Ambiguity in Defining Advanced Gastric Cancer

Within our analysis, we recognize the absence of a standardized definition for advanced gastric cancer and acknowledge the potential implications of this ambiguity on the interpretation and generalizability of our findings.

#### 3.3.1. Lack of Standardized Definition

Defining advanced gastric cancer proves challenging due to its complexity and heterogeneity. While various classification systems exist, such as TNM staging and the Japanese Gastric Cancer Association (JGCA) classifications, a universally accepted definition remains elusive due to variations in tumor extent, lymph node involvement, and other prognostic factors.

#### 3.3.2. Implications for Interpretation

The lack of a standardized definition for advanced gastric cancer complicates the interpretation and comparability of findings across studies. The potential implications of this ambiguity are as follows:

1. Variability in Patient Selection

The absence of a standardized definition can lead to variations in patient selection, with some studies including patients with earlier-stage disease, while others focus exclusively on advanced cases. This variation can introduce heterogeneity and limit the generalizability of the results.

2. Confounding Factors

The lack of a standardized definition may result in different baseline characteristics of patients across studies, including variations in tumor location, histology, and comorbidities. These factors can influence treatment outcomes and confound the assessment of the impact of lymphadenectomy.

3. Treatment Strategies

The definition of advanced gastric cancer can influence treatment strategies, including the extent of lymphadenectomy. Without a consistent definition, different surgical approaches and lymph node dissection techniques may be utilized, further complicating the comparison of outcomes across studies.

4. Reporting Bias

The absence of a standardized definition could contribute to reporting bias. Studies may selectively report outcomes that align with their specific definition of advanced disease, potentially skewing the overall interpretation of the impact of lymphadenectomy.

#### 3.3.3. Overcoming Ambiguity

To address the ambiguity in defining advanced gastric cancer, future research should strive towards developing and adopting a standardized definition. Establishing a consensus on the extent of tumor invasion, lymph node involvement, and other relevant prognostic factors is crucial. This approach would facilitate the comparability of studies, improve the understanding of treatment outcomes, and enhance evidence-based decision making.

In conclusion, the lack of a standardized definition for advanced gastric cancer poses challenges in interpreting and generalizing the findings regarding the impact of lymphadenectomy. Acknowledging this ambiguity and its potential implications is essential in critically evaluating the evidence. Future studies should aim to establish a consensus definition, thereby improving the comparability and reliability of research findings in this field.

### 3.4. Lymphadenectomy in Advanced Gastric Cancer: Rationale, Significance and Clinical Implications 

Lymphadenectomy is a crucial procedure in the surgical treatment of advanced gastric cancer. It is widely used because lymph nodes play a significant role in the spread of cancer, and removing them can improve outcomes for patients with advanced disease [23,24].

The lymphatic system transports lymph, a fluid containing white blood cells, throughout the body. Lymph nodes act as filters for the lymphatic system, capturing harmful substances like bacteria, viruses, and cancer cells. When cancer cells break away from the primary tumor, they can travel through the lymphatic system and become trapped in nearby lymph nodes. From there, they can grow and spread to other parts of the body [25].

There are two main reasons for performing lymphadenectomy in advanced gastric cancer. Firstly, it provides information about the stage of the disease and the extent of cancer spread. The number, location, and size of lymph nodes affected by cancer are essential factors in determining the stage of the cancer. This information helps in planning further treatment and prognosis. Secondly, lymphadenectomy helps reduce the risk of cancer recurrence. Cancer cells can remain dormant in the lymph nodes for a long time before becoming active again. If these cells are not removed during surgery, they can potentially cause the cancer to return. Removing the affected lymph nodes eliminates any cancer cells that may have spread and reduces the risk of recurrence.

The spread of cancer through the lymphatic system is a key mechanism by which gastric cancer can metastasize to distant organs. Lymphadenectomy aims to remove these potentially cancerous nodes before they can spread the disease.

In advanced gastric cancer, removing lymph nodes can help prevent the spread of cancer cells to other parts of the body. The lymph nodes near the stomach are often the first places that cancer cells travel to. By removing these lymph nodes, surgeons can eliminate any cancer cells that may have started to grow there, potentially prolonging the patient’s life. Recent studies have shown that lymphadenectomy can improve outcomes for patients with advanced gastric cancer. A 2019 meta-analysis of 16 studies found that lymphadenectomy was associated with improved overall survival and disease-free survival, particularly in patients with more advanced stages of the disease [26].

### 3.5. Exploring Lymphadenectomy in Gastric Cancer: A Comparative Analysis of Eastern and Western Approaches

In Eastern countries, such as Japan and South Korea, a more extensive lymphadenectomy technique known as D2 or D2+ dissection is commonly practiced. This approach aims to remove a larger number of lymph nodes in order to eliminate potential metastatic disease and improve locoregional control. Eastern surgeons believe that by removing a greater number of lymph nodes, they can increase the chances of complete tumor resection [27,28,29].

On the other hand, Western countries, including the United States and European nations, generally adopt less extensive lymphadenectomy techniques, such as D1 or D1+ dissections [30]. The rationale behind these approaches is to strike a balance between achieving optimal oncologic outcomes and minimizing surgery-related morbidity. Western surgeons often rely on systemic chemotherapy to manage potential undetected metastases, leading them to prioritize minimizing surgical morbidity.

The variations in lymphadenectomy techniques between Eastern and Western countries can be influenced by multiple factors. Patient characteristics, tumor biology, surgical expertise, and even cultural factors can contribute to these differences. Additionally, historical practice patterns and disparities in healthcare systems and resources may also play a role.

In summary, lymphadenectomy in gastric cancer differs between Eastern and Western countries. The former tend to adopt more extensive techniques, while the latter prefer less extensive approaches. Collaborative efforts and a global perspective are essential to improve our understanding of optimal lymphadenectomy strategies. By analyzing and synthesizing outcomes from both regions, clinicians can make informed decisions tailored to each patient’s individual characteristics and cultural context.

### 3.6. The Significance of Subdivision into Stations during Lymphadenectomy in Gastric Cancer Management 

Lymphadenectomy in gastric cancer involves the removal of lymph nodes around the stomach to evaluate and treat potential metastases. In recent decades, two main approaches, D1+ and D2+, have been developed and utilized in practice.

The D1+ approach involves the removal of lymph nodes at stations 1–7. This includes the removal of lymph nodes at the gastric and perigastric lymph nodes, as well as the splenic and hepatoduodenal lymph nodes. This approach is considered more conservative and less invasive than D2+, but it may be associated with a higher risk of lymphatic recurrence [31]. 

In contrast, the D2+ approach involves the removal of lymph nodes at stations 1–16. This includes stations 8–12, which represent the supraduodenal perigastric lymph nodes and lymph nodes at the level of the common hepatic and splenic arteries [31]. This approach is considered more radical and comprehensive in terms of lymph node removal, aiming to treat potential metastases and reduce the risk of lymphatic recurrence.

The subdivision into stations during lymphadenectomy in gastric cancer is important to more accurately assess the extent and implications of lymph node metastases. It provides a detailed description of the location and degree of lymph node involvement, which can impact prognosis and treatment choice. For example, harvesting lymph nodes at station 11, which involves lymph nodes at the level of the common hepatic artery, may be relevant in the decision to remove the spleen if there is tumor invasion in that area.

In conclusion, the subdivision into stations during lymphadenectomy in gastric cancer, in both the D1+ and D2+ approaches, is essential for accurately assessing the extent of the cancer and planning appropriate treatment. It provides crucial information for therapeutic planning and achieving optimal outcomes in gastric cancer management.

### 3.7. Overview of D1+ and D2+ Lymphadenectomy 

The management of gastric cancer patients involves two different surgical techniques: D1+ lymphadenectomy (removal of the primary tumor and nearby lymph nodes) and D2+ lymphadenectomy (removal of a broader range of lymph nodes, including those near the main arteries and veins near the stomach).

D1+ lymphadenectomy primarily focuses on accurate tumor staging and reducing locoregional recurrences, while D2+ lymphadenectomy is a more extensive procedure that includes the removal of a wider range of lymph nodes. This technique aims to remove more lymph nodes that may potentially harbor metastatic tumor cells.

Research studies have compared the outcomes of D1+ and D2+ lymphadenectomy to evaluate their effectiveness and safety. One study found no significant difference in overall survival between the two techniques [32], suggesting that D1+ lymphadenectomy may suffice for certain patients. However, another study reported improved overall survival rates with D2+ lymphadenectomy [33], suggesting potential benefits.

Despite these findings, there are ongoing controversies regarding the optimal extent of lymphadenectomy. Concerns have been raised about increased operative complications associated with D2+ lymphadenectomy, such as pancreatic leakage or splenic infarction.

In conclusion, D1+ and D2+ lymphadenectomy are distinct surgical approaches used in treating gastric cancer. The choice between these techniques depends on various factors and remains a subject of debate. Further research is needed to better understand the advantages and complications of each approach and establish standardized guidelines for lymphadenectomy in gastric cancer.

### 3.8. D3 Lymphadenectomy

#### 3.8.1. D3 Lymphadenectomy Procedure

D3 lymphadenectomy is a surgical procedure performed in gastric cancer treatment to remove lymph nodes in specific anatomical regions. The objective of this procedure is to achieve an extended lymph node dissection, allowing for accurate staging and the potential increase in chances of removing involved lymph nodes [34].

The D3 lymphadenectomy procedure involves the removal of lymph nodes from designated stations, including groups 1 (perigastric), 2 (behind the stomach), 3 (suprapancreatic), 4 (left gastric artery), 7 (hepatoduodenal ligament), 8a (common hepatic artery), 9 (celiac axis), and 11p (splenic hilum). During surgery, meticulous dissection along anatomical landmarks is performed to identify and remove lymph nodes in these regions.

#### 3.8.2. Techniques Employed

Various techniques are employed in D3 lymphadenectomy to ensure a thorough lymph node dissection while preserving important structures. These techniques include meticulous dissection along the anatomical planes; the careful identification and preservation of major vessels, such as the left gastric artery, common hepatic artery, and celiac axis; and attention to nerve preservation to minimize postoperative morbidity [34].

Experienced surgeons play a crucial role in performing D3 lymphadenectomy as they possess the necessary expertise to navigate complex anatomical structures and ensure the safety and adequacy of the procedure.

#### 3.8.3. Associated Outcomes

D3 lymphadenectomy has been associated with several favorable outcomes in gastric cancer treatment [34]:

(a) Improved Staging Accuracy: D3 lymphadenectomy provides a more extensive lymph node dissection, leading to improved accuracy in cancer staging. Accurate staging helps guide treatment decisions and predict patient prognosis.
(b) Increased Lymph Node Yield: Compared to less extensive lymphadenectomy procedures, D3 lymphadenectomy yields a higher number of harvested lymph nodes. This increased lymph node yield improves the likelihood of identifying involved lymph nodes and facilitates precise pathological staging.
(c) Enhanced Disease Control: D3 lymphadenectomy aims to remove lymph nodes that are at higher risk of containing metastatic cancer cells. By removing a more extensive lymph node basin, D3 lymphadenectomy potentially improves disease control and reduces the risk of locoregional recurrence.
(d) Potential Survival Benefits: Several studies have suggested that D3 lymphadenectomy may confer survival benefits in gastric cancer patients. These benefits may be attributed to accurate staging, increased lymph node yield, and improved disease control.

#### 3.8.4. The Effectiveness and Benefits of D3 Lymphadenectomy

Recent studies have evaluated the effectiveness and benefits of D3 lymphadenectomy in gastric cancer treatment:

(a) JCOG9501: This Japanese study compared D2 lymphadenectomy (removal of groups 1–7 lymph nodes) with D2+ lymphadenectomy (removal of groups 8a, 9, and 11p in addition to D2 lymphadenectomy). Results showed that D2+ lymphadenectomy improved overall survival and reduced locoregional recurrence compared to D2 lymphadenectomy [35].
(b) CLASS-01 Trial: This Chinese study compared the survival outcomes and surgical morbidity between D2 and D2+ lymphadenectomy. The results demonstrated that D2+ lymphadenectomy provided superior 3-year survival rates and lower recurrence rates compared to D2 lymphadenectomy, with acceptable surgical morbidity [36].
(c) ARTIST Trial: This Korean study compared a D2+ lymphadenectomy with adjuvant chemotherapy to a D2 lymphadenectomy with adjuvant chemotherapy in patients with stage II-III gastric cancer [37]. The study showed no significant difference in overall survival between the two groups, suggesting that adjuvant chemotherapy may have a greater impact on survival than the extent of lymphadenectomy.

These recent studies highlight the potential effectiveness and benefits of D3 lymphadenectomy, including improved survival outcomes and reduced recurrence rates. However, it is worth noting that the optimal extent of lymphadenectomy may vary depending on patient characteristics, surgical expertise, and institutional guidelines.

### 3.9. Emerging Technologies in Lymphadenectomy for Advanced Gastric Cancer

#### 3.9.1. Minimally Invasive Approaches: Laparoscopic and Robotic Surgery

Laparoscopic and robotic surgery are minimally invasive approaches that have been increasingly utilized in the management of gastric cancer. Compared to open surgery, these approaches have been associated with reduced blood loss, fewer complications, and shorter hospital stays. Several studies have reported equivalent oncological outcomes between laparoscopic/robotic and open surgery in patients with advanced gastric cancer [38]. 

One of the most promising approaches is laparoscopic lymphadenectomy, which involves the use of a tiny camera and instruments inserted through small incisions in the abdomen. This technique has been shown to be as effective as open lymphadenectomy in terms of survival and recurrence rates, while causing less pain, faster recovery, and shorter hospital stays [39]. Robotic-assisted lymphadenectomy is another minimally invasive option that uses a robotic arm to manipulate the surgical instruments. This approach allows for greater precision and ease of movement, increasing the surgeon’s ability to navigate complex anatomy and to perform more extensive lymph node removal [40]. 

#### 3.9.2. Imaging Techniques

Preoperative imaging techniques, such as computed tomography (CT) and magnetic resonance imaging (MRI), are primary tools for evaluating lymph node metastasis. However, these imaging modalities have limited sensitivity for detecting microscopic lymph node metastasis. Several new imaging techniques have been developed to improve the accuracy of detecting lymph node metastasis, including endoscopic ultrasound (EUS) and positron emission tomography (PET) [41]. 

Imaging techniques have also been developed to improve the accuracy of lymphadenectomy. One such approach is indocyanine green (ICG) fluorescence imaging, which involves injecting a fluorescent dye that is absorbed by lymph nodes. The dye can then be detected using a special camera to identify the location and number of affected nodes. This technique has been shown to be effective in guiding lymphadenectomy in gastric cancer patients, as well as identifying lymph node metastases in other types of cancer.

#### 3.9.3. Biomarker-Guided Approaches

Biomarkers, such as circulating tumor cells (CTCs), tumor-associated macrophages (TAMs), and microRNAs, have been proposed as new tools for identifying lymph node metastasis. For example, the presence of circulating tumor cells (CTCs) in the peripheral blood has been associated with poor outcomes in gastric cancer patients, suggesting their potential as a biomarker for lymph node metastasis [42]. Similarly, tumor-associated macrophages (TAMs) have been shown to promote tumor progression and metastasis, and their presence in resected lymph nodes has been associated with a poor prognosis [43].

Biomarker-guided approaches represent another promising strategy for lymphadenectomy in advanced gastric cancer (AGC). By analyzing genetic and molecular changes in cancer cells, biomarkers can be used to identify and target specific types of cancer cells. For example, the biomarker HER2 has been found to be overexpressed in a subset of gastric cancer patients, and targeted therapy with trastuzumab has been shown to improve survival in this patient population. Similarly, biomarkers, such as PD-L1 and microsatellite instability (MSI), can be used to guide the use of immunotherapy in gastric cancer patients.

### 3.10. The Role of Lymphadenectomy in the Management of Advanced Gastric Cancer

Lymphadenectomy serves as a vital cornerstone in the management of advanced gastric cancer, as it allows for accurate staging by providing critical information about the extent of disease spread. By removing involved lymph nodes, it helps to prevent further metastatic dissemination. Additionally, lymphadenectomies enable pathologists to ascertain the metastatic burden, aiding in prognostic determinations and guiding the selection of adjuvant therapies. 

Several research studies, randomized controlled trials (RCTs), and observational studies have investigated the role of lymphadenectomy in gastric cancer treatment. A meta-analysis of 11 RCTs by Guo et al. (2020) found that extended lymphadenectomy was associated with an overall survival (OS) benefit compared to limited lymphadenectomy [44]. Furthermore, the study showed that the survival advantage was significant in patients with locally advanced gastric cancer. Similarly, a retrospective study of 1267 gastric cancer patients by Liu et al. (2020) found that extended lymphadenectomy could improve OS in patients with advanced gastric cancer [45].

Various studies have revealed the therapeutic benefits of lymphadenectomy alongside primary tumor resection. Extensive regional lymph node dissection (D2 lymphadenectomy) has been associated with improved overall survival rates in certain patient populations, particularly in Asian countries [46]. However, it is important to consider that the effectiveness of lymphadenectomy may vary based on factors such as tumor stage, location, histological type, and the presence of distant metastasis.

Clinical trials and observational studies have shown the benefits of lymphadenectomy in patients with gastric cancer [47,48]. In a meta-analysis of 32 studies, Zhang et al. (2019) showed that lymphadenectomy improved overall survival and disease-free survival in patients with gastric cancer [49]. The study included a total of 23,831 patients and reported a significant benefit in 5-year overall survival with extended lymphadenectomy compared to standard lymphadenectomy (hazard ratio (HR) = 0.84, 95% confidence interval (CI) 0.77–0.91).

However, some studies have also suggested that lymphadenectomy may not always be necessary or beneficial for patients with advanced gastric cancer, particularly in cases where the cancer has spread to other organs or the lymph nodes are too difficult to access. A study published in the European Journal of Surgical Oncology in 2020 found that lymphadenectomy did not improve survival rates for patients with advanced gastric cancer who underwent palliative surgery [50].

The benefits of lymphadenectomy must be weighed against potential risks and complications (Table 1 and Table 2). 

### 3.11. The Effectiveness of D1 Lymphadenectomy Compared to D2 Lymphadenectomy in Treating Advanced Gastric Cancer

In order to assess the effectiveness of different lymphadenectomy procedures, it is crucial to understand the rationale behind D1 and D2 lymphadenectomy, their respective techniques, and the associated outcomes.

D1 lymphadenectomy entails the surgical removal of lymph nodes in the perigastric area, specifically around the stomach (including the lesser and greater curvature), along the left gastric artery up to the splenic hilum. This procedure offers the advantage of lowered morbidity and mortality rates compared to more extensive lymphadenectomy techniques. In contrast, the D2 lymphadenectomy involves a more radical approach, including the comprehensive removal of lymph nodes in the perigastric region, along the celiac axis, splenic artery, hepatic artery, and the retropancreatic region. The primary aim of D2 lymphadenectomy is to eliminate an extended spectrum of potentially affected lymph nodes and reduce the chance of residual tumor burden.

Advocates of D1 lymphadenectomy argue that it is a less invasive procedure, resulting in fewer surgical complications and a shorter hospital stay. Consequently, postoperative recovery is generally faster, allowing patients to regain their normal activities sooner. Additionally, proponents assert that D1 lymphadenectomy demonstrates comparable long-term survival rates to D2 lymphadenectomy, particularly in patients with early-stage gastric cancer. This argument is supported by studies indicating that for T1 and T2 lesions, D1 lymphadenectomy offers satisfactory oncological outcomes with relatively low rates of tumor recurrence.

On the other hand, proponents of D2 lymphadenectomy argue that its more extensive removal of lymph nodes results in a decreased risk of locoregional recurrence and improved disease-free survival rates. They assert that D2 lymphadenectomy provides a more thorough staging of the disease, enabling accurate patient prognosis and the identification of suitable candidates for adjuvant therapies. Furthermore, proponents believe that D2 lymphadenectomy is especially beneficial for patients with advanced gastric cancer and those at higher risk of lymph node metastasis, as it enables more effective eradication of undetected micrometastases and eliminates any potential residual tumor burden.

Despite the aforementioned arguments supporting both approaches, the evidence for the superiority of D2 lymphadenectomy in all advanced gastric cancer cases is inconclusive. Several large-scale randomized controlled trials, such as the Dutch Gastric Cancer Group (DGCG) trial and the British Stomach Cancer Group (BSCG) trial, have shown conflicting results regarding the survival benefits of D2 lymphadenectomy [62,63]. These studies reported either limited or no significant differences in postoperative outcomes and overall survival rates between D1 and D2 lymphadenectomy groups.

Based on a systematic review conducted by the Cochrane Library in 2018, D2 lymphadenectomy is considered the standard surgical treatment for advanced gastric cancer [64]. This is because D2 lymphadenectomy is associated with better overall survival rates compared to D1 lymphadenectomy. This systematic review examines the evidence for the effectiveness and safety of D2 lymphadenectomy compared to D1 lymphadenectomy in the treatment of gastric cancer. The review includes 22 studies with a total of 7805 participants, all of whom had gastric cancer and underwent either D1 or D2 lymphadenectomy. The primary outcome was overall survival, and secondary outcomes included disease-free survival, operative mortality, and morbidity.

The results of the review indicate that D2 lymphadenectomy improves overall survival and disease-free survival compared to D1 lymphadenectomy. However, D2 lymphadenectomy is associated with higher operative mortality and morbidity, including a higher risk of pancreatic fistula and delayed gastric emptying. The review suggests that D2 lymphadenectomy is a more effective treatment for gastric cancer than D1 lymphadenectomy, but it also carries some risks. The decision to perform D2 lymphadenectomy should be made on a case-by-case basis, taking into account the patient’s individual risk factors and preferences.

Overall, while some studies suggest that D2 lymphadenectomy may be more effective than D1 lymphadenectomy in terms of lymph node retrieval and tumor recurrence, there is still no clear evidence that one procedure is superior in terms of overall survival. 

Therefore, the selection of the lymphadenectomy approach should be tailored to the individual needs of each patient, taking into account the patient’s stage of disease, overall health, and the surgeon’s expertise.

Ultimately, the decision to perform a D1 or D2 lymphadenectomy will depend on a variety of factors, including the stage of the cancer, the patient’s overall health, and the preference of the surgeon and medical team.

### 3.12. Controversy Surrounding Lymphadenectomy in Advanced Gastric Cancer: Insights from Clinical Trials and the Current Standard of Care

The controversy surrounding lymphadenectomy in treating advanced gastric cancer has provided valuable insights into the importance of personalized treatment plans. As the field continues to evolve, healthcare providers must stay updated with the latest evidence and collaborate with patients to make informed treatment decisions.

The primary source of controversy lies in the lack of consensus regarding the extent and number of lymph nodes to be removed during surgery. This uncertainty has led to a debate on the efficacy of extensive lymphadenectomy versus lymphatic mapping and targeted removal of specific lymph nodes. Complicating matters further is the fact that studies investigating the effectiveness of lymphadenectomy in advanced gastric cancer have often produced conflicting results.

Recent studies have supported the argument for D2 lymphadenectomy in the management of advanced gastric cancer. For example, a notable meta-analysis of randomized clinical trials found a positive association between D2 lymphadenectomy and improved overall survival compared to less extensive procedures [65]. Additionally, a national study from Sweden demonstrated that patients who underwent D2 lymphadenectomy had significantly enhanced overall survival rates compared to those who underwent D1 lymphadenectomy [66].

However, it is important to note that numerous studies and clinical trials investigating the use of lymphadenectomy in advanced gastric cancer have yielded varying findings. While some studies indicate a potential improvement in survival rates, others show no significant benefits or even showed increased complications associated with the procedure [67,68,69,70,71,72,73,74,75]. These conflicting results underscore the need for further research and the development of personalized treatment plans based on individual patient characteristics.

Overall, the controversy surrounding lymphadenectomy in advanced gastric cancer highlights the need for personalized treatment plans and the continuous evaluation of the latest evidence. By staying informed and collaborating with patients, healthcare providers can make better-informed decisions to optimize outcomes for patients with advanced gastric cancer.

### 3.13. Comparative Analysis of Lymphadenectomy Approaches in Advanced Gastric Cancer: Balancing Benefits, Risks, and Impact on Postoperative Complications and Quality of Life

Lymphadenectomy serves as a vital tool in cancer management, enabling accurate staging and improving the chances of a successful cure. However, the decision to undergo this procedure requires careful consideration of the potential benefits and risks. 

#### 3.13.1. Benefits of Lymphadenectomy

1. Enhanced Cancer Staging: By removing lymph nodes, surgeons can accurately assess the extent of cancer and its spread to neighboring tissues or organs. This precise staging assists in developing appropriate treatment plans and predicting patient prognosis.
2. Increased Cure Rates: In cases where the cancer is localized within the lymph nodes, complete removal may eliminate the cancer entirely. Moreover, combining lymphadenectomy with complementary treatments, like chemotherapy or radiation therapy, can significantly improve the chances of a successful outcome.

#### 3.13.2. The Benefits and Effectiveness of D1 Lymphadenectomy

D1 lymphadenectomy encompasses the removal of primary gastric tumor, along with a limited number of regional lymph nodes [52]. As a less aggressive approach, D1 lymphadenectomy is associated with certain advantages. Firstly, it allows for shorter operative times, thereby minimizing the risk of intraoperative complications and reducing patient discomfort. Furthermore, studies have reported lower rates of postoperative complications, such as anastomotic leakage and infection, following D1 lymphadenectomy. While this technique may result in a reduced number of lymph nodes excised, it has demonstrated similar overall survival rates when compared to D2 dissections in specific patient cohorts. Thus, D1 lymphadenectomy can be deemed as a feasible alternative with favorable outcomes for carefully selected patients.

#### 3.13.3. The Benefits and Effectiveness of D2 Lymphadenectomy

D2 lymphadenectomy involves the removal of an extended set of regional lymph nodes, ensuring a more comprehensive excision. This approach provides several benefits for patients with advanced gastric cancer [76,77]. Firstly, D2 lymphadenectomy increases the likelihood of detecting and removing metastatic lymph nodes, improving staging accuracy and subsequent treatment decisions. Additionally, it has been linked to a reduction in locoregional tumor recurrence rates and improved disease-free survival when compared to D1 lymphadenectomy. Despite the longer operative times and increased risk of complications associated with D2 lymphadenectomy, studies have shown that these risks can be effectively managed with careful surgical expertise and postoperative care [78,79,80]. Overall, D2 lymphadenectomy offers superior oncological outcomes, particularly for patients at high risk of disease progression.

#### 3.13.4. Risks and Complications of Lymphadenectomy [58,80,81]

1. Bleeding and Infection: Like any surgical procedure, lymphadenectomy carries a risk of bleeding and infection. These complications, if not promptly addressed, can lead to adverse health effects and prolong the recovery process.
2. Damage to Surrounding Structures: The proximity of lymph nodes to vital structures raises the possibility of inadvertent damage to organs or tissues during the procedure. Such damage can pose severe health risks and necessitate additional interventions.

D1 versus D2 lymphadenectomy can significantly influence postoperative complications and quality of life for patients undergoing treatment for advanced gastric cancer. 

### 3.14. D2 versus D1 Lymphadenectomy Postoperative Complications

When comparing the postoperative complications specifically between D2 and D1 lymphadenectomy in gastric cancer, studies have shown that D2 lymphadenectomy carries a higher risk [80,82,83,84]. Several complications have been reported more frequently following D2 lymphadenectomy.

One of the most commonly observed postoperative complications after D2 lymphadenectomy is an anastomotic leakage. The risk of anastomotic leakage is generally higher with D2 lymphadenectomy due to the extensive dissection and increased complexity of the procedure.

Pancreatic fistula is another complication that tends to occur more frequently with D2 lymphadenectomy. It involves the leakage of pancreatic fluids from the surgical site. The removal of more lymph nodes and closer proximity to the pancreas in D2 versus D1 lymphadenectomy can increase the likelihood of this complication.

Intra-abdominal abscesses, which are localized collections of pus within the abdominal cavity, are also reported as more common after D2 lymphadenectomy. The extensive dissection performed during D2 lymphadenectomy can lead to infections and subsequent abscess formation.

Wound infections, including superficial and deep surgical site infections, are seen with a slightly higher incidence following D2 lymphadenectomy. The large incisions made during the procedure provide more opportunities for bacterial colonization and infection.

It is important to note that the risk and severity of complications can vary depending on individual patient factors and surgical techniques employed. Skilled surgeons who perform D2 lymphadenectomy in high-volume centers may have lower complication rates, emphasizing the importance of experience and expertise in minimizing adverse outcomes.

In conclusion, while D2 lymphadenectomy might offer better oncological outcomes for patients with advanced gastric cancer, it is associated with a higher risk of postoperative complications, including anastomotic leakage and delayed gastric emptying, as well as decreased quality of life compared to D1 lymphadenectomy. Therefore, healthcare professionals should carefully consider the potential benefits and risks of D2 lymphadenectomy before deciding on the appropriate surgical approach for patients with advanced gastric cancer.

### 3.15. Impact of Neoadjuvant Treatment on Lymphadenectomy Outcomes in Advanced Gastric Cancer

Neoadjuvant treatment is an increasingly utilized strategy in the management of advanced gastric cancer [85]. 

Neoadjuvant treatment, consisting of chemotherapy, chemoradiotherapy, or a combination, has emerged as a valuable strategy to improve outcomes in these patients. Lymphadenectomy, the removal of regional lymph nodes during surgical resection, is an essential component of curative treatment for gastric cancer patients. However, the impact of neoadjuvant treatment on lymphadenectomy outcomes is still a subject of debate.

#### 3.15.1. Effects of Neoadjuvant Treatment on Lymph Node Yield

Recent studies have investigated the association between neoadjuvant treatment and lymph node yield during surgery [85,86,87]. While some studies have reported a decreased lymph node yield in patients receiving neoadjuvant treatment, others have shown no significant difference compared to patients undergoing upfront surgery. Variations in study design, patient selection, and neoadjuvant treatment regimens may partially explain these discrepancies.

#### 3.15.2. Pathological Response and Survival Rates

Neoadjuvant treatment is known to induce pathological responses, characterized by tumor regression and fibrotic changes, which can be evaluated through histological examination. Several studies have demonstrated a correlation between better pathological response and improved survival outcomes in patients receiving neoadjuvant treatment. Furthermore, extensive lymphadenectomy in combination with neoadjuvant treatment has been associated with increased survival rates, suggesting that the two interventions may have a synergistic effect.

#### 3.15.3. Recommendations and Guidelines

Current guidelines recommend extended lymphadenectomy in cases of locally advanced gastric cancer. However, the impact of neoadjuvant treatment on lymphadenectomy extent is yet to be fully elucidated. Studies have shown that patients undergoing neoadjuvant treatment may have more limited lymph node involvement, potentially questioning the necessity of extensive lymphadenectomy in these cases. Further research is warranted to establish clear guidelines regarding lymphadenectomy extent in the context of neoadjuvant treatment.

Neoadjuvant treatment plays a crucial role in the management of advanced gastric cancer. While the impact of neoadjuvant treatment on lymphadenectomy outcomes is still debated, evidence suggests that neoadjuvant treatment can induce favorable pathological responses and improve survival rates, particularly when combined with extensive lymphadenectomy. The extent of lymphadenectomy in the context of neoadjuvant treatment remains an area of ongoing research, and future studies are needed to further elucidate the optimal approach in these patients.

### 3.16. Comparing Futures: Ongoing vs. Concluded Clinical Trials

Clinical trials are an important part of evaluating medical interventions. There are two main types of trials: ongoing and closed. While assessing the impact of lymphadenectomy in gastric cancer, both ongoing and closed trials have provided valuable insights. Ongoing trials collect real-time data and assess current treatment strategies. This allows researchers to observe outcomes and continually evaluate the effectiveness and safety of lymphadenectomy. Closed trials, on the other hand, have completed data collection and provide comprehensive analysis and results. They explore various aspects related to lymphadenectomy, such as survival rates and complications. Ongoing trials offer real-time insights, while closed trials provide valuable data for future clinical decision making. It is important to assess multiple trial results collectively to obtain a comprehensive understanding. Meta-analyses and systematic reviews help summarize findings.

## 4. Conclusions and Future Directions

Lymphadenectomy plays a critical role in the management of advanced gastric cancer by removing cancerous lymph nodes and improving survival rates. However, determining the optimal extent of lymph node dissection remains a topic of ongoing debate. High levels of lymph node removal have been associated with increased risks of complications and morbidity.

Current evidence indicates that extended lymphadenectomy may be beneficial for patients with locally advanced cancer or those at high risk of lymph node metastasis. On the other hand, limited lymphadenectomy has shown comparable outcomes and fewer complications in low-risk patients. Therefore, the selection of the appropriate lymphadenectomy extent should be based on the patient’s risk profile and the surgeon’s experience.

To ensure the safety and efficacy of lymphadenectomy, surgeons should undergo adequate training and possess expertise in the technique. The multidisciplinary approach should be adopted for the optimal management of advanced gastric cancer, involving the evaluation of lymph node status and the selection of the appropriate extent of lymphadenectomy based on tumor staging.

It is important to evaluate the risk–benefit ratio of lymphadenectomy on an individual basis, taking into consideration the patient’s preoperative status, comorbidities, tumor characteristics and the surgeon’s operative skill. This personalized approach will help minimize complications and maximize the potential benefits of the procedure.

Further research is needed to determine the optimal extent of lymphadenectomy and identify specific patient subgroups that may derive the greatest benefit from this procedure. Until more evidence is available, clinicians should carefully weigh the potential benefits and risks of lymphadenectomy on a case-by-case basis, considering individual patient characteristics and preferences.

Lymphadenectomy remains an essential component in the management of advanced gastric cancer, providing valuable staging information and influencing treatment decisions. While there is conflicting evidence regarding its efficacy, lymphadenectomy remains a valuable tool for both diagnosis and treatment. By individualizing the approach based on patient characteristics and surgeon experience, we can enhance the safety and effectiveness of lymphadenectomy in the context of advanced gastric cancer.

Future directions in lymphadenectomy for advanced gastric cancer may involve refining the extent of lymphadenectomy based on individualized treatment approaches. This can be achieved through the use of preoperative imaging to better identify high-risk patients who would benefit from more extensive lymphadenectomy. Furthermore, incorporating techniques such as sentinel lymph node biopsy can help reduce the extent of lymphadenectomy in lower-risk patients.

In terms of future perspectives and research directions, there is a need to identify biomarkers that can guide patient selection for lymphadenectomy. This will enable a more targeted approach to treatment. Additionally, novel techniques that improve the effectiveness of lymphadenectomy while minimizing the risks of complications should be explored. Furthermore, ongoing research in immunotherapy and other systemic treatments for advanced gastric cancer may provide alternative options for patients who are not suitable candidates for surgery. Ultimately, achieving optimal outcomes in patients with advanced gastric cancer will require a personalized approach based on individual patient characteristics and tumor biology.

Another area of research interest is the use of precision medicine approaches for gastric cancer treatment. This involves identifying specific genetic or molecular alterations in a patient’s tumor, which can then be targeted by specific drugs or therapies. Clinical trials evaluating the effectiveness of targeted therapies for gastric cancer are currently underway, and further research in this area may lead to personalized treatment options for patients with advanced disease.

Additionally, there is a need for further research into the optimal timing of lymphadenectomy, particularly in the context of neoadjuvant and adjuvant therapy. Neoadjuvant therapy may potentially improve the success of lymphadenectomy by reducing tumor burden and enhancing overall patient outcomes.

In conclusion, while lymphadenectomy remains an important component of treatment for advanced gastric cancer, ongoing research is essential to optimize its use and minimize associated morbidity. This includes refining the extent of lymphadenectomy through individualized treatment approaches, identifying biomarkers to guide patient selection, exploring novel techniques to improve effectiveness, and investigating precision medicine approaches. Additionally, further research into the optimal timing of lymphadenectomy is needed in the context of neoadjuvant and adjuvant therapy. By continually advancing our understanding and refining our approach, we can ultimately achieve better outcomes for patients with advanced gastric cancer.

## Figures and Tables

**Table 1:** Comparison of D1 and D2 lymphadenectomy approaches in advanced gastric cancer [51,52,53,54,55,56,57,58].

Characteristics	D1 Lymphadenectomy	D2 Lymphadenectomy
Extent of lymph node dissection	Removal of perigastric lymph nodes (stations 1–6)	Removal of perigastric and extragastric lymph nodes (stations 1–12)
Number of retrieved lymph nodes	Average of 15–25	Average of 30–40
Operative time	Generally shorter (around 2–4 h)	Generally longer (around 4–6 h)
Postoperative complications	Lower risk of complications such as anastomotic leakage or pancreatic fistula Lower risk of delayed gastric emptying	Higher risk of complications such as anastomotic leakage or pancreatic fistula Higher risk of delayed gastric emptying Higher risk of intra-abdominal abscess Higher risk of lymphatic leakage Higher risk of bleeding Higher risk of wound infection

(Table 1 provides a concise summary of the key characteristics and differences between D1 and D2 lymphadenectomy approaches in terms of the extent of lymph node dissection, number of retrieved lymph nodes, operative time, and postoperative complications) [51,52,53,54,55,56,57,58].

**Table 2:** Comparison of short-term outcomes: D1 lymphadenectomy vs. D2 lymphadenectomy in gastric cancer treatment [51,59,60,61,62].

Short-Term Outcomes	D1 Lymphadenectomy	D2 Lymphadenectomy
Length of Hospital Stay (days)	8–14	12–16
Postoperative Morbidity (%)	20–30	25–35
Mortality Rate (%)	1–5	2–7

(Table 2 provides a general overview of the short-term outcomes, but it is important to note that these ranges may vary depending on several factors, such as the patient’s overall health, tumor stage, and surgical skill. Additionally, these short-term outcomes provide a general overview and should be considered in conjunction with long-term outcomes and other factors when making treatment decisions for gastric cancer patients) [51,59,60,61,62].

## Supplementary Materials

The following supporting information can be downloaded at: https://www.mdpi.com/article/10.3390/life13081769/s1, The supplementary material titled "PRISMA Flow Diagram" provides a visual representation of the study selection process. It illustrates the screening, eligibility, and selection criteria followed during the literature review for this study. This diagram offers a clear overview of the included and excluded studies, ensuring transparency and replicability in our methodology.

## References

[1] Martin D.N., Lam T.K., Brignole K., Ashing K.T., Blot W.J., Burhansstipanov L., Chen J.T., Dignan M., Gomez S.L., Martinez M.E. (2016). Recommendations for Cancer Epidemiologic Research in Understudied Populations and Implications for Future Needs. Cancer Epidemiol. Biomark. Prev..

[2] World Health Organization (2021). Cancer. https://www.who.int/news-room/fact-sheets/detail/cancer.

[3] American Cancer Society Cancer Facts & Figures 2021. https://www.cancer.org/content/dam/cancer-org/research/cancer-facts-and-statistics/annual-cancer-facts-and-figures/2021/cancer-facts-and-figures-2021.pdf.

[4] Bray F., Ferlay J., Soerjomataram I., Siegel R.L., Torre L.A., Jemal A. (2018). Global cancer statistics 2018: GLOBOCAN estimates of incidence and mortality worldwide for 36 cancers in 185 countries. CA Cancer J. Clin..

[5] NCCN Guidelines Version 4.2019 Gastric Cancer. https://www.nccn.org/professionals/physician_gls/pdf/gastric.pdf.

[6] World Cancer Research Fund International (2021). Cancer Statistics. https://www.wcrf.org/dietandcancer/cancer-trends/worldwide-cancer-data.

[7] International Agency for Research on Cancer (2020). GLOBOCAN 2020: Romania Fact Sheet. https://gco.iarc.fr/today/data/factsheets/populations/642-romania-fact-sheets.pdf.

[8] Machlowska J., Baj J., Sitarz M., Maciejewski R., Sitarz R. (2020). Gastric Cancer: Epidemiology, Risk Factors, Classification, Genomic Characteristics and Treatment Strategies. Int. J. Mol. Sci..

[9] Eslick G.D. (2006). Helicobacter pylori infection causes gastric cancer? A review of the epidemiological, meta-analytic, and experimental evidence. World J. Gastroenterol..

[10] Rawla P., Barsouk A. (2019). Epidemiology of gastric cancer: Global trends, risk factors and prevention. Przegląd Gastroenterol..

[11] Sexton R.E., Al Hallak M.N., Diab M., Azmi A.S. (2020). Gastric cancer: A comprehensive review of current and future treatment strategies. Cancer Metastasis Rev..

[12] Wang P.L., Xiao F.T., Gong B.C., Liu F.N. (2017). Alcohol drinking and gastric cancer risk: A meta-analysis of observational studies. Oncotarget.

[13] Fang X., Wei J., He X., An P., Wang H., Jiang L., Shao D., Liang H., Li Y., Wang F. (2015). Landscape of dietary factors associated with risk of gastric cancer: A systematic review and dose-response meta-analysis of prospective cohort studies. Eur. J. Cancer.

[14] Tian J., Liu G., Zuo C., Liu C., He W., Chen H. (2019). Genetic polymorphisms and gastric cancer risk: A comprehensive review synopsis from meta-analysis and genome-wide association studies. Cancer Biol. Med..

[15] Choi Y.J., Kim N. (2016). Gastric cancer and family history. Korean J. Intern. Med..

[16] World Health Organization: International Agency for Research on Cancer (IARC) Globocan 2020—Stomach Cancer. https://gco.iarc.fr/today/data/factsheets/cancers/7-Stomach-fact-sheet.pdf.

[17] National Institute of Diabetes and Digestive and Kidney Diseases (NIDDK) Stomach Cancer. https://www.niddk.nih.gov/health-information/digestive-diseases/stomach-cancer#causes.

[18] Mayo Clinic Gastric Cancer. https://www.mayoclinic.org/diseases-conditions/gastric-cancer/symptoms-causes/syc-20352688.

[19] American Society of Clinical Oncology Stomach (Gastric) Cancer—Introduction. https://www.cancer.net/cancer-types/stomach-gastric-cancer/introduction.

[20] Marano L., Carbone L., Poto G.E., Restaino V., Piccioni S.A., Verre L., Roviello F., Marrelli D. (2023). Extended Lymphadenectomy for Gastric Cancer in the Neoadjuvant Era: Current Status, Clinical Implications and Contentious Issues. Curr. Oncol..

[21] Tamura S., Takeno A., Miki H. (2011). Lymph node dissection in curative gastrectomy for advanced gastric cancer. Int. J. Surg. Oncol..

[22] Japanese Gastric Cancer Association (2020). Japanese gastric cancer treatment guidelines 2018 (5th edition). Gastric Cancer.

[23] Garg P.K., Jakhetiya A., Sharma J., Ray M.D., Pandey D. (2016). Lymphadenectomy in gastric cancer: Contentious issues. World J. Gastrointest. Surg..

[24] Proserpio I., Rausei S., Barzaghi S., Frattini F., Galli F., Iovino D., Rovera F., Boni L., Dionigi G., Pinotti G. (2014). Multimodal treatment of gastric cancer. World J. Gastrointest. Surg..

[25] Miftode S., Bruns H. (2022). Misclassification of nodal stage in gastric cancer: 16 lymph nodes is not enough. Surg. Exp. Pathol..

[26] Ferro A., Peleteiro B., Malvezzi M., Bosetti C., Bertuccio P., Levi F., Negri E., La Vecchia C., Lunet N. (2019). Global cancer incidence and mortality for gastric cancer: A systematic review and meta-analysis of global patterns and trends. Gut.

[27] Wang X., Zhao J., Shen Z., Fairweather M., Enzinger P.C., Sun Y., Wang J. (2020). Multidisciplinary Approach in Improving Survival Outcome of Early-Stage Gastric Cancer. J. Surg. Res..

[28] Zhang Y.X., Yang K. (2020). Significance of nodal dissection and nodal positivity in gastric cancer. Transl. Gastroenterol. Hepatol..

[29] Strong V.E., Yoon S.S. (2013). Extended lymphadenectomy in gastric cancer is debatable. World J. Surg..

[30] Yoon S.S., Yang H.K. (2009). Lymphadenectomy for gastric adenocarcinoma: Should west meet east?. Oncologist.

[31] Degiuli M., De Manzoni G., Di Leo A., D’Ugo D., Galasso E., Marrelli D., Petrioli R., Polom K., Roviello F., Santullo F. (2016). Gastric cancer: Current status of lymph node dissection. World J. Gastroenterol..

[32] Bonenkamp J.J., Hermans J., Sasako M., Welvaart K., Songun I., Meyer S., Plukker J.T.M., Van Elk P., Obertop H., Gouma D.J. (1999). Extended lymph-node dissection for gastric cancer. N. Engl. J. Med..

[33] Sano T., Sasako M., Mizusawa J., Yamamoto S., Katai H., Yoshikawa T., Nashimoto A., Ito S., Kaji M., Imamura H. (2017). Randomized Controlled Trial to Evaluate Splenectomy in Total Gastrectomy for Proximal Gastric Carcinoma. Ann. Surg..

[34] Douridas G.N., Pierrakakis S.K. (2018). Is There Any Role for D3 Lymphadenectomy in Gastric Cancer?. Front. Surg..

[35] Sasako M., Sano T., Yamamoto S., Kurokawa Y., Nashimoto A., Kurita A., Hiratsuka M., Tsujinaka T., Kinoshita T., Arai K. (2008). D2 lymphadenectomy alone or with para-aortic nodal dissection for gastric cancer. N. Engl. J. Med..

[36] Yu J., Huang C., Sun Y., Su X., Cao H., Hu J., Wang K., Suo J., Tao K., He X. (2019). Effect of Laparoscopic vs Open Distal Gastrectomy on 3-Year Disease-Free Survival in Patients With Locally Advanced Gastric Cancer: The CLASS-01 Randomized Clinical Trial. JAMA.

[37] Lee J., Lim D.H., Kim S., Park S.H., Park J.O., Park Y.S., Lim H.Y., Choi M.G., Sohn T.S., Noh J.H. (2012). Phase III trial comparing capecitabine plus cisplatin versus capecitabine plus cisplatin with concurrent capecitabine radiotherapy in completely resected gastric cancer with D2 lymph node dissection: The ARTIST trial. J. Clin. Oncol..

[38] Chen Q.Y., Xie J.W., Zhong Q., Wang J.B., Lin J.X., Lu J., Cao L.L., Lin M., Tu R.H., Huang Z.N. (2020). Safety and Efficacy of Indocyanine Green Tracer-Guided Lymph Node Dissection During Laparoscopic Radical Gastrectomy in Patients With Gastric Cancer: A Randomized Clinical Trial. JAMA Surg..

[39] He H., Li H., Su X., Li Z., Yu P., Huang H., Huang C., Ye J., Li Y., Suo J. (2018). Study on safety of laparoscopic total gastrectomy for clinical stage I gastric cancer: The protocol of the CLASS02-01 multicenter randomized controlled clinical trial. BMC Cancer.

[40] Uyama I., Suda K., Nakauchi M., Kinoshita T., Noshiro H., Takiguchi S., Ehara K., Obama K., Kuwabara S., Okabe H. (2019). Clinical advantages of robotic gastrectomy for clinical stage I/II gastric cancer: A multi-institutional prospective single-arm study. Gastric Cancer.

[41] Kumar D., Pande G., Agarwal A., Bhalla A.S., Sharma R. (2020). Diagnostic accuracy of PET/CT compared with MRI and CT in the detection of gastric cancer recurrence. Abdom. Radiol..

[42] Son T., Lee J.W., Kim Y.W., Hyung W.J., Kim H.-I. (2016). Prognostic significance of circulating tumor cells in patients with locally advanced gastric cancer who underwent gastrectomy: A prospective study. Ann. Surg. Oncol..

[43] Oh S.-Y., Kim Y.S., Choi Y.-J., Kim K.M., Jeon H.M. (2018). Tumor-associated macrophages (TAMs) in colon cancer and their clinical implications. Arch. Pathol. Lab. Med..

[44] Guo J., Li B., Liu Y., Dai R., Yuan Y., Chen L., Wang G. (2020). Extended lymphadenectomy for resectable gastric cancer: A systematic review and meta-analysis of randomized controlled trials. World J. Surg. Oncol..

[45] Liu M., Feng F., Guo M. (2020). Lymphadenectomy for gastric cancer: Should D2 dissection be the standard?. J. Gastroenterol..

[46] Chen K., Sun B., Liang Y., Yin Y., Chen C., Wang S., Deng S., Wang C., Cai W. (2020). D2 versus D1 gastrectomy for advanced gastric cancer: A retrospective cohort study. Int. J. Surg..

[47] Li D., Xing X., Wang X., Li Z., Zhang T., Fang M., Li Y. (2020). Benefit of lymphadenectomy for advanced gastric cancer patients with metastasis to the lymph nodes in the gastrohepatic ligament or celiac region. Front. Oncol..

[48] Kim H., Woo Y., Kim Y. (2020). Lymphadenectomy for advanced gastric cancer in the era of minimally invasive surgery and precision medicine. Ann. Surg. Oncol..

[49] Zhang T.Y., Duan H.Q., Zhou Z.X., Zhao Y.P., Zhang X.M., Zhang W.H., Zhang Y. (2020). Comparison of laparoscopic versus open radical lymphadenectomy for gastric cancer with respect to postoperative complications and oncologic outcomes: A propensity score analysis. Surg. Endosc..

[50] Huerta S., Cappello J., Oh S. (2020). Lymphadenectomy in palliative gastrectomy for stage IV gastric cancer. Eur. J. Surg. Oncol..

[51] Songun I., Putter H., Kranenbarg E.M., Sasako M., van de Velde C.J. (2010). Surgical treatment of gastric cancer: 15-year follow-up results of the randomised nationwide Dutch D1D2 trial. Lancet Oncol..

[52] Schmidt B., Yoon S.S. (2013). D1 versus D2 lymphadenectomy for gastric cancer. J. Surg. Oncol..

[53] Memon M.A., Subramanya M.S., Khan S., Hossain M.B., Osland E., Memon B. (2011). Meta-analysis of D1 versus D2 gastrectomy for gastric adenocarcinoma. Ann. Surg..

[54] Di Martino N., Izzo G., Cosenza A., Vicenzo L., Monaco L., Torelli F., Basciotti A., Brillantino A., Marra A. (2005). Gastrectomia totale per cancro gastrico: Può il tipo di linfadenectomia condizionare i risultati a distanza? [Total gastrectomy for gastric cancer: Can the type of lymphadenectomy condition the long-term results?]. Suppl. Tumori.

[55] Lavy R., Hershkovitz Y., Chikman B., Shapira Z., Poluksht N., Yarom N., Sandbank J., Halevy A. (2015). D1 versus D2 Gastrectomy for Gastric Adenocarcinoma. Isr. Med. Assoc. J..

[56] Kang J.H., Ryu S.Y., Jung M.R., Jeong O. (2020). Comparison of long term survival outcomes between D1+ and D2 lymph node dissection for ≥pT2 or pN+ gastric carcinoma: A large scale case-control study using propensity score matching. Eur. J. Surg. Oncol..

[57] Luo X., Zhou M.X., Tian W., Zeng M., Xia J.L., Zhao G.P., Hu H.L., Hao X.B., Han L.F., Liu H. (2020). A retrospective study comparing D1 limited lymph node dissection and D2 extended lymph node dissection for N3 gastric cancer. Transl. Cancer Res..

[58] Lam S., Tan E., Menezes A., Martin D., Gallagher J., Storey D., Sandroussi C. (2018). A comparison of the operative outcomes of D1 and D2 gastrectomy performed at a single Western center with multiple surgeons: A retrospective analysis with propensity score matching. World J. Surg. Oncol..

[59] Seevaratnam R., Bocicariu A., Cardoso R., Mahar A., Kiss A., Helyer L., Law C. (2012). A meta-analysis of D1 versus D2 lymph node dissection. Gastric Cancer.

[60] Degiuli M., Sasako M., Calgaro M., Garino M., Rebecchi F., Mineccia M., Scaglione D., Andreone D., Ponti A., Calvo F. (2004). Morbidity and mortality after D1 and D2 gastrectomy for cancer: Interim analysis of the Italian Gastric Cancer Study Group (IGCSG) randomised surgical trial. Eur. J. Surg. Oncol..

[61] Ruiz E., Sanchez J., Celis J., Payet E., Berrospi F., Chavez I., Young F. (2009). Cancer gastrico localizado: Resultados quirurgicos de 801 pacientes tratados con linfadenectomia d2 [Surgical outcome of 801 patients with localized gastric cancer treated with d2 lymphadenectomy]. Rev. Gastroenterol. Peru.

[62] Songun I., Keizer H.J., Hermans J., Klementschitsch P., de Vries J.E., Wils J.A., van der Bijl J., van Krieken J.H., van de Velde C.J. (1999). Chemotherapy for operable gastric cancer: Results of the Dutch randomised FAMTX trial. The Dutch Gastric Cancer Group (DGCG). Eur. J. Cancer.

[63] Allum W.H., Hallissey M.T., Ward L.C., Hockey M.S. (1989). A controlled, prospective, randomised trial of adjuvant chemotherapy or radiotherapy in resectable gastric cancer: Interim report. British Stomach Cancer Group. Br. J. Cancer.

[64] Cochrane Library (2018). D2 Versus D1 Lymphadenectomy for Gastric Cancer. https://www.cochranelibrary.com/cdsr/doi/10.1002/14651858.CD00786.pub2/full.

[65] Degiuli M., Reddavid R., Tomatis M., Ponti A., Morino M., Sasako M., Rebecchi F., Garino M., Vigano L., Scaglione D. (2021). D2 dissection improves disease-specific survival in advanced gastric cancer patients: 15-year follow-up results of the Italian Gastric Cancer Study Group D1 versus D2 randomised controlled trial. Eur. J. Cancer.

[66] Kung C.H., Tsai J.A., Lundell L., Johansson J., Nilsson M., Lindblad M. (2020). Nationwide study of the impact of D2 lymphadenectomy on survival after gastric cancer surgery. BJS Open.

[67] Yashiro M., Matsuoka T. (2015). Sentinel node navigation surgery for gastric cancer: Overview and perspective. World J. Gastrointest. Surg..

[68] Hiratsuka M., Miyashiro I., Ishikawa O., Furukawa H., Motomura K., Ohigashi H., Kameyama M., Sasaki Y., Kabuto T., Ishiguro S. (2001). Application of sentinel node biopsy to gastric cancer surgery. Surgery.

[69] Maruyama K., Gunvén P., Okabayashi K., Sasako M., Kinoshita T. (1989). Lymph node metastases of gastric cancer. General pattern in 1931 patients. Ann. Surg..

[70] Amin M.B., Greene F.L., Edge S.B., Compton C.C., Gershenwald J.E., Brookland R.K., Meyer L., Gress D.M., Byrd D.R., Winchester D.P. (2017). The Eighth Edition AJCC Cancer Staging Manual: Continuing to build a bridge from a population-based to a more “personalized” approach to cancer staging. CA Cancer J. Clin..

[71] Sano T., Coit D.G., Kim H.H., Roviello F., Kassab P., Wittekind C., Yamamoto Y., Ohashi Y. (2017). Proposal of a new stage grouping of gastric cancer for TNM classification: International Gastric Cancer Association staging project. Gastric Cancer.

[72] Cuschieri A., Weeden S., Fielding J., Bancewicz J., Craven J., Joypaul V., Sydes M., Fayers P. (1999). Patient survival after D1 and D2 resections for gastric cancer: Long-term results of the MRC randomized surgical trial. Surgical Co-operative Group. Br. J. Cancer.

[73] Hartgrink H.H., van de Velde C.J., Putter H., Bonenkamp J.J., Klein Kranenbarg E., Songun I., Welvaart K., van Krieken J.H., Meijer S., Plukker J.T. (2004). Extended lymph node dissection for gastric cancer: Who may benefit? Final results of the randomized Dutch gastric cancer group trial. J. Clin. Oncol..

[74] Kurokawa Y., Sasako M., Sano T., Yoshikawa T., Iwasaki Y., Nashimoto A., Ito S., Kurita A., Mizusawa J., Nakamura K. (2015). Ten-year follow-up results of a randomized clinical trial comparing left thoracoabdominal and abdominal transhiatal approaches to total gastrectomy for adenocarcinoma of the oesophagogastric junction or gastric cardia. Br. J. Surg..

[75] Lee H.J., Hyung W.J., Yang H.K., Han S.U., Park Y.K., An J.Y., Kim W., Kim H.I., Kim H.H., Ryu S.W. (2019). Short-term Outcomes of a Multicenter Randomized Controlled Trial Comparing Laparoscopic Distal Gastrectomy with D2 Lymphadenectomy to Open Distal Gastrectomy for Locally Advanced Gastric Cancer (KLASS-02-RCT). Ann. Surg..

[76] Karavokyros I., Michalinos A. (2018). Favoring D2-Lymphadenectomy in Gastric Cancer. Front. Surg..

[77] Shen J., Cao B., Wang Y., Xiao A., Qin J., Wu J., Yan Q., Hu Y., Yang C., Cao Z. (2018). Prospective randomized controlled trial to compare laparoscopic distal gastrectomy (D2 lymphadenectomy plus complete mesogastrium excision, D2 + CME) with conventional D2 lymphadenectomy for locally advanced gastric adenocarcinoma: Study protocol for a randomized controlled trial. Trials.

[78] Weledji E.P. (2017). The principles of the surgical management of gastric cancer. Int. J. Surg. Oncol..

[79] Cao B., Xiao A., Shen J., Xie D., Gong J. (2020). An Optimal Surgical Approach for Suprapancreatic Area Dissection in Laparoscopic D2 Gastrectomy with Complete Mesogastric Excision. J. Gastrointest. Surg..

[80] Barchi L.C., Charruf A.Z., de Oliveira R.J., Jacob C.E., Cecconello I., Zilberstein B. (2016). Management of postoperative complications of lymphadenectomy. Transl. Gastroenterol. Hepatol..

[81] Kodera Y., Sasako M., Yamamoto S., Sano T., Nashimoto A., Kurita A., Gastric Cancer Surgery Study Group of Japan Clinical Oncology Group (2005). Identification of risk factors for the development of complications following extended and superextended lymphadenectomies for gastric cancer. Br. J. Surg..

[82] Kulig J., Popiela T., Kolodziejczyk P., Sierzega M., Szczepanik A., Polish Gastric Cancer Study Group (2007). Standard D2 versus extended D2 (D2+) lymphadenectomy for gastric cancer: An interim safety analysis of a multicenter, randomized, clinical trial. Am. J. Surg..

[83] Danielson H., Kokkola A., Kiviluoto T., Sirén J., Louhimo J., Kivilaakso E., Puolakkainen P. (2007). Clinical outcome after D1 vs D2-3 gastrectomy for treatment of gastric cancer. Scand. J. Surg..

[84] Rausei S., Ruspi L., Rosa F., Morgagni P., Marrelli D., Cossu A., Cananzi F.C., Lomonaco R., Coniglio A., Biondi A. (2016). Extended lymphadenectomy in elderly and/or highly co-morbid gastric cancer patients: A retrospective multicenter study. Eur. J. Surg. Oncol..

[85] Yan Y., Yang A., Lu L., Zhao Z., Li C., Li W., Chao J., Liu T., Fong Y., Fu W. (2021). Impact of Neoadjuvant Therapy on Minimally Invasive Surgical Outcomes in Advanced Gastric Cancer: An International Propensity Score-Matched Study. Ann. Surg. Oncol..

[86] Reddavid R., Sofia S., Chiaro P., Colli F., Trapani R., Esposito L., Solej M., Degiuli M. (2018). Neoadjuvant chemotherapy for gastric cancer. Is it a must or a fake?. World J. Gastroenterol..

[87] Xu A.M., Huang L., Liu W., Gao S., Han W.X., Wei Z.J. (2014). Neoadjuvant chemotherapy followed by surgery versus surgery alone for gastric carcinoma: Systematic review and meta-analysis of randomized controlled trials. PLoS ONE.

