# Supplementary material for: Uncovering the Impact of Lymphadenectomy in Advanced Gastric Cancer: A Comprehensive Review
Source: Life (Basel). 2023 Aug 18;13(8):1769. doi: 10.3390/life13081769 (PMC10455758; doi:10.3390/life13081769)
Supplement: Supplementary file 1 [file life-13-01769-s001.zip › life-2528895-supplementary.pdf]

PRISMA 2020 flow diagram for new systematic reviews which included searches of databases and registers only

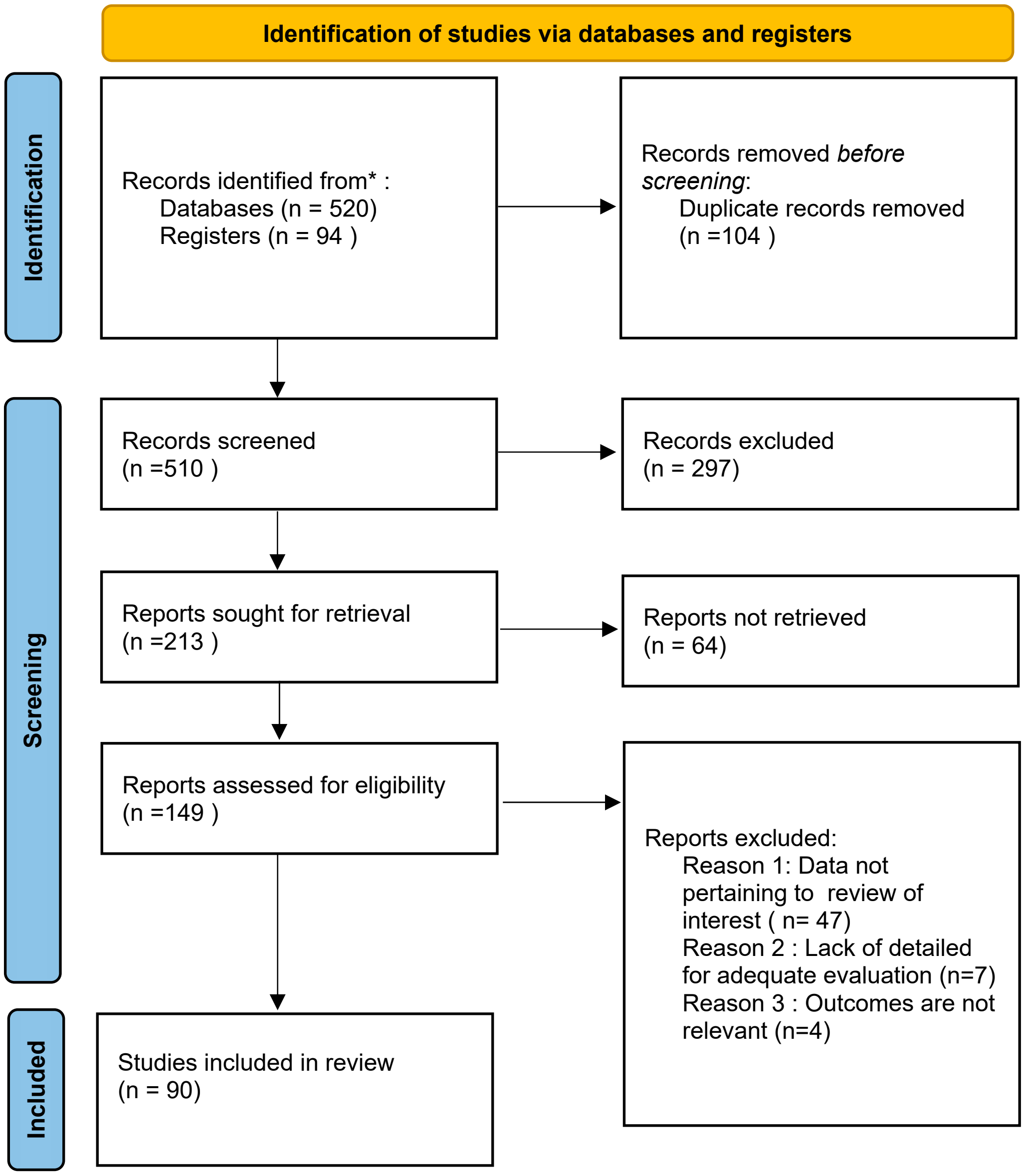

*Consider, if feasible to do so, reporting the number of records identified from each database or register searched (rather than the total number across all databases/registers).

From: Page MJ, McKenzie JE, Bossuyt PM, Boutron I, Hoffmann TC, Mulrow CD, et al. The PRISMA 2020 statement: an updated guideline for reporting systematic reviews. BMJ 2021;372:n71. doi: 10.1136/bmj.n71

For more information, visit: http://www.prisma-statement.org/
